# Synaptonemal Complex Components Promote Centromere Pairing in Pre-meiotic Germ Cells

**DOI:** 10.1371/journal.pgen.1004012

**Published:** 2013-12-19

**Authors:** Nicolas Christophorou, Thomas Rubin, Jean-René Huynh

**Affiliations:** 1 Department of Genetics and Developmental Biology, Institut Curie, Paris, France; 2 CNRS UMR3215; Inserm U934, Paris, France; Stowers Institute for Medical Research, United States of America

## Abstract

Mitosis and meiosis are two distinct cell division programs. During mitosis, sister chromatids separate, whereas during the first meiotic division, homologous chromosomes pair and then segregate from each other. In most organisms, germ cells do both programs sequentially, as they first amplify through mitosis, before switching to meiosis to produce haploid gametes. Here, we show that autosomal chromosomes are unpaired at their centromeres in *Drosophila* germline stem cells, and become paired during the following four mitosis of the differentiating daughter cell. Surprisingly, we further demonstrate that components of the central region of the synaptonemal complex are already expressed in the mitotic region of the ovaries, localize close to centromeres, and promote *de novo* association of centromeres. Our results thus show that meiotic proteins and meiotic organization of centromeres, which are key features to ensure reductional segregation, are laid out in amplifying germ cells, before meiosis has started.

## Author Summary

Meiosis is a special type of cell division occurring in germ cells to produce sexual gametes. Initially, germ cells contain two copies of each chromosome, one from the mother and one from the father, which are called homologs. During meiosis, cells divide twice to produce haploid gametes with only one copy of each chromosome. Each gamete receives exactly one copy of each chromosome, because homologs become associated, through a process called meiotic pairing, and then segregate from each other during the first round of division. In *Drosophila*, it was assumed that homologs were always paired in every cell type. Meiotic pairing was thus viewed as an extension of a pre-existing pairing. Here, we show that chromosomes II and III are not paired in germline stem cells, which produce germ cells throughout adult life. We further show that these chromosomes become paired during the four rounds of mitosis preceding the entry in meiosis. Surprisingly, meiotic proteins are expressed during these four rounds of mitosis and are required for homologs to pair. Our results thus show that, in *Drosophila*, meiosis starts during the preceding mitosis.

## Introduction

**Figure 1:**
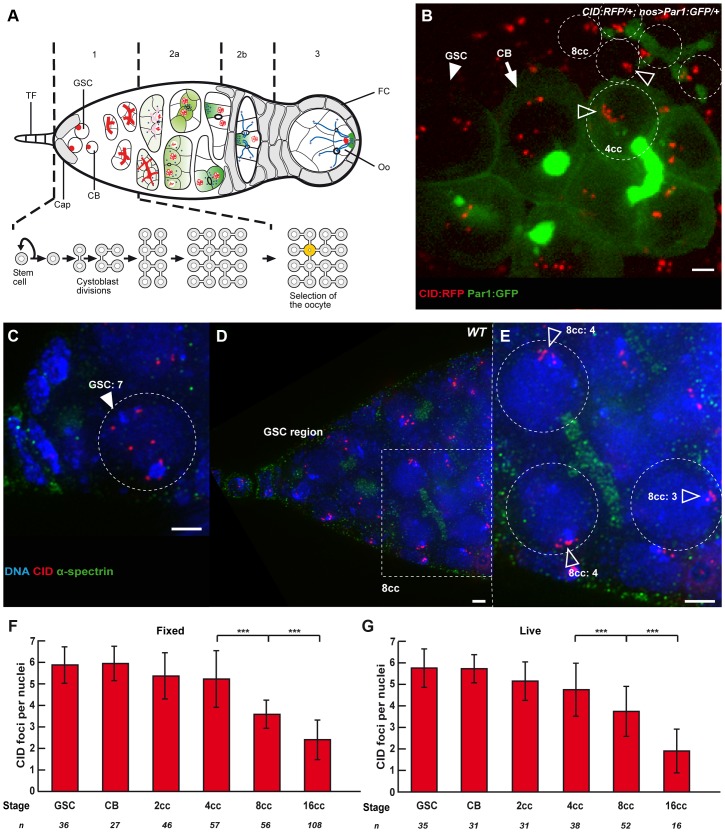
Dispersed centromeres in germline stem cells become paired during cyst divisions. (A) A schematic for developmental stages during Drosophila female meiosis. The egg chambers are produced in a specialized structure, called the germarium, at the anterior of the ovariole. The germarium is divided into four morphological regions along the anterior–posterior axis. The germline stem cells (GSCs) reside at the anterior tip of the germarium (left) and divide to produce cystoblasts, which divide four more times in region 1 to produce 16 cell germline cysts that are connected by ring canals. The stem cells and cystoblasts (CB) contain a spectrosome (red circles), which develops into a branched structure called the fusome, which orients each division of the cyst. In early region 2a, the synaptonemal complex (red lines) forms along the chromosomes of the two cells with four ring canals (pro-oocytes, yellow) as they enter meiosis. The synaptonemal complex then appears transiently in the two cells with three ring canals, before becoming restricted to the pro-oocytes in late region 2a. By region 2b, the oocyte (Oo) has been selected, and is the only cell to remain in meiosis. Terminal Filament (TF), Cap cells (Cap), Follicle Cells (FC). Blue dots are centrosomes migrating into the oocyte. Green shade shows the progressive restriction of some proteins or mRNAs into the oocyte. (B) Projection of Z-sections obtained by time lapse microscopy (spinning disc) of a living CID::RFP/+; nos>Par1::GFP/+ germarium expressing the centromere marker CID::RFP (red) and the fusome marker Par1::GFP (green). One Germline Stem Cell (GSC, arrowhead) is identified by its position close to the cap cells (not shown), one cystoblast (CB, arrow) is identified by a round bright fusome, and a 4-cell cyst (4cc), whose cells are linked by a typical U-shaped fusome, demonstrating that they are from the same cyst. One nucleus of a 4-cell cyst (4cc) and 5 nuclei of an 8-cell cyst (8cc), with fusome GFP, are surrounded by dotted lines. Open arrows indicate centromeres. Scale bar represents 2 µm. (C–E) C is a projection of z-sections, D and E are single sections, all obtained by DV microscopy of a wild-type fixed germarium stained for centromere (CID, red), fusome (α-spectrin, green), and DNA (DAPI, blue). Close up on a GSC (C, GSC) and on 3 nuclei of an 8-cell cyst (E, 8cc) from the corresponding boxed region in D are shown. 7 centromeres can be distinguished in the GSC, while centromeres are clustered (open arrows) in 8-cell cyst nuclei. The number of distinguishable centromeres is indicated for each 8cc nucleus. Note that the GSC close up in C includes more projected images than the corresponding area in D to show the exact number of centromeres. Scale bars represent 2 µm. (F,G) Developmental changes in the number of CID foci for each cell stage in region 1 in fixed wild-type germaria (F) or in living germaria (G). The number of analyzed cells is indicated under each stage. *** p≤0.0005 (two-tailed Student's t-test comparing 4cc with 8cc and 8cc with 16cc).

In *Drosophila* females, mitosis and meiosis occur sequentially throughout adult life in two distinct regions of the germarium at the tip of each ovary [1]. In the mitotic zone (called region 1), germline stem cells (GSCs) generate a precursor cell called a cystoblast (CB), which undergoes exactly four mitoses to produce a germline cyst made of 16 cells (Figure 1A). These mitoses are not complete and all 16 sister cells remain connected through ring canals and by an organelle called the fusome, which links all cells. The branched shape of the fusome is a useful marker to distinguish each stage, GSC, CB, 2-, 4-, 8- and 16-cell cyst (cc) in the mitotic zone [2]. After the last mitosis, cysts enter region 2 and all 16 cells start meiosis [3]. During differentiation in region 2, only one cell per cyst, however, remains in meiosis, while the 15 others exit meiosis and endoreplicate their DNA [4].

How homologous chromosomes find each other is a central question in reproductive biology. In most organisms, this process involves pairing of homologous chromosomes, formation of the synaptonemal complex (SC), genetic recombination and formation of crossing-over [5]–[6]. In flies, homologous chromosomes are always paired in somatic cells, a phenomenon called “somatic pairing” [7]. Meiotic pairing is thus viewed as an extension of a pre-existing somatic pairing. Live-imaging provided strong support for this hypothesis in males, and in females, it is known that homologous chromosomes are already paired when cysts enter region 2 [8]–[12]. Recently, an additional organization of meiotic chromosomes was described in *Drosophila* females, whereby centromeres of all chromosomes aggregate into one or two clusters [13]–[14]. This organization is reminiscent of the clustering of telomeres during the bouquet stage in other species. It was further shown that formation of the synaptonemal complex (synapsis) initiates at centromere clusters, and that SC components, such as C(3)G and Corona, were required for centromere clustering in addition to synapsis [13]–[14]. In this study, we set out to explore the organization of chromosomes in the earliest stages of adult germ cell development before the onset of meiosis.

## Results

### Unpaired centromeres in germline stem cells

To distinguish the different stages of region 1, we marked the fusome with an antibody against α-spectrin, a membrane skeletal protein, and to visualize centromeres, we used an anti-CID antibody marking the *Drosophila* homologue of Cenp-A, a histone H3 variant present only in centromeric regions [15] (Figure 1C–E). *Drosophila* diploid cells have eight chromosomes representing 4 pairs of homologues, therefore when homologues are all paired, one should distinguish 4 dots of CID. When centromeres are not all paired, one should see more than 4 dots, and if centromeres are clustered, one should see 1 or 2 dots [13]–[14]. We used the same methodology as published in (Takeo et al., 2011). CID foci were scored as unpaired as soon as a single “black” pixel could be distinguished between the two foci. Overlapping foci were all considered paired. We found in GSCs and CBs an average of 6 dots of CID (GSCs: 5.9±0.8, n = 36; CBs: 5.9±0.8, n = 27), which indicates that centromeres were not all paired (Figure 1F). This result is in contrast with most cell types examined so far by us and others in *Drosophila* males or females. We also found that centromeres became more associated, as cysts underwent more divisions. Indeed, 2-cell cysts showed an average of 5.3±1.1 CID foci, 4-cell cysts 5.2±1.3 CID foci and it dropped to 3.6±1.3 dots of CID in 8-cell cysts and 2.4±0.9 in 16-cell cysts (Figure 1F). Thus, there was a decrease of almost 50% in the number of CID dots from GSCs to 8-cell cysts, indicating that most 8-cell cysts had already paired their centromeres in the mitotic region. We also observed the appearance of classes of nuclei with only 1 or 2 dots of CID in 8-cell cysts (19,6%, n = 56 ; Figure S1A) indicating a tendency for centromeres to cluster already at this stage. To confirm that our fixation procedure preserved the 3D organization of nuclei in the germarium, we repeated our experiments using live-imaging on intact tissue with CID-RFP and Par-1-GFP to label the fusome (Figure 1B). We found the same numbers of CID dots at every stage confirming our previous results with fixed tissue (Figure 1G). We further found that centromere movements were highly dynamic whether as single, paired or clustered centromeres (Movie S1). Even when centromeres were clustered, it was not a static nor passive maintenance of a Rabl organization [16]. We concluded that several centromeres were unpaired in GSCs, but that most centromeres became associated before the onset of meiosis.

### Centromeres of chromosome II and III become associated in the mitotic region, whereas X-chromosome centromeres are always paired

**Figure 2:**
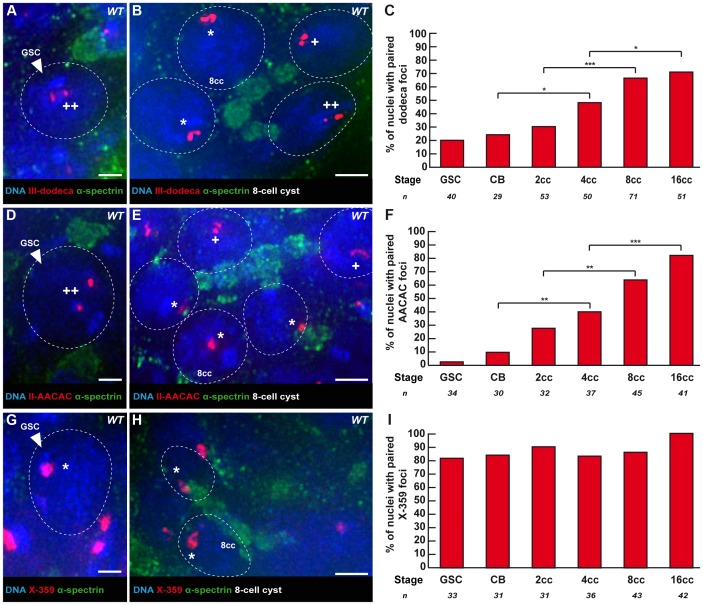
Centromeres of chromosome II and III become associated in the mitotic region, whereas X-chromosome centromeres are always paired. Fusome (α-spectrin, green) and DNA (DAPI, blue). (A, B) Projection of Z-sections obtained by DV microscopy of a wild-type germarium stained for the chromosome III centromere (dodeca probe, red), fusome, and DNA. (A) GSC showing 2 dodeca foci separated by a distance >0,7 µm. (B) 4 nuclei of a 8-cell cyst showing foci separated by a distance <0,7 µm. Nuclei with * show one focus, thus centromeres are paired, nuclei with one + display two foci separated by a distance ≤0,7 µm thus they are considered paired and nuclei with ++ display two foci separated by a distance >0,7 µm and are thus considered unpaired. Scale bars represent 2 µm. (C) Developmental changes in the percentage of paired chromosome III centromeres for each cell stage in region 1 using the dodeca probe. The number of cells analyzed is indicated under each stage. * p≤0.05, ** p≤0.005, *** p≤0.0005 (khi2 test comparing CB with 4cc, 2cc with 8cc and 4cc with 16cc). (D, E) Projection of Z-sections obtained by DV microscopy of a wild-type germarium stained for the chromosome II centromere (AACAC probe, red), fusome, and DNA. (D) GSC showing 2 AACAC foci separated by a distance >0,7 µm. (E) 5 nuclei of a 8-cell cyst showing foci separated by a distance <0,7 µm. Nuclei with * show one foci, thus centromeres are paired, nuclei with one + display two foci separated by a distance ≤0,7 µm thus they are considered paired and nuclei with ++ display two foci separated by a distance >0,7 µm and are thus considered unpaired. Scale bars represent 2 µm. (F) Developmental changes in the percentage of paired chromosome II centromeres for each cell stage in region 1 using the AACAC probe. The number of cells analyzed is indicated under each stage. * p≤0.05, ** p≤0.005, *** p≤0.0005 (khi2 test comparing CB with 4cc, 2cc with 8cc and 4cc with 16cc). (G, H) Projection of Z-sections obtained by DV microscopy of a wild-type germarium stained for the chromosome X centromere (359 probe, red), fusome, and DNA. (G) GSC showing one 359 focus. (H) 2 nuclei of an 8-cell cyst showing paired foci. Nuclei with * show one focus, thus centromeres are paired. Scale bars represent 2 µm. (I) Developmental changes in the percentage of paired X-chromosome centromeres for each cell stage in region 1 using the 359 probe. The number of cells analyzed is indicated under each stage.

We next investigated the pairing behaviour of individual chromosomes to test if pre-meiotic centromere pairing occurred between homologous chromosomes. We used the dodeca, the AACAC and the 359 probes to label the pericentromeric regions of chromosome *III*, *II* and *X*, respectively [17]. We combined fluorescence in situ hybridisation (FISH) with immunostaining against the fusome marker α-spectrin (Figure 2). We considered that chromosomes were paired when only one focus was visible or when two foci were visible separated by a distance ≤0.70 µm [9]–[10]. We found that the peri-centromeric region of chromosome *III* and *II* became paired individually in region 1, with the same timing as observed with the centromere marker CID. In GSCs and CBs only 20.0% (n = 40) and 24.1% (n = 29) of nuclei displayed pairing of the peri-centromeric region of chromosome *III* (Figure 2A, C). The peri-centromeric region of chromosome *II* was paired in only 2.9% (n = 34) of GSCs and 10.0% (n = 30) of CBs (Figure 2D, F). In 8-cell cysts 66.2% (n = 71) of nuclei displayed pairing with the dodeca probe, and 71% (n = 51) in 16-cell cysts (Figure 2B, C). Similarly, with the AACAC probe, 64.4% (n = 45) of 8-cell cysts were paired, and 82.9% (n = 41) of 16-cell cysts (Figure 2E, F). In contrast, the peri-centromeric region of the *X* chromosome appeared mostly paired already in GSCs (82%, n = 33), and only slightly increased to 86% (n = 43) of pairing in 8-cell cysts and 100% (n = 42) of pairing in 16-cell cysts (Figure 2G–I). We thus concluded that while the *X*-chromosome peri-centromeric region is always paired, there is *de novo* pairing for centromeres of chromosome *II* and *III* in the mitotic region.

As indicated previously, we found that all centromeres showed a tendency to cluster already in 8-cell cysts (Figure S1A), raising the possibility of interactions between centromeres of non-homologous chromosomes. We thus measured for each centromere the percentage of interaction with a non-homologous centromere at each stage. At the 8-cell stage, for example, we found significant interactions between centromeres of chromosome III and X (28.7%, n = 108 and 38%, n = 108; Figure S2A and S2C). These percentages were, however, well below homologous interactions at the same stage (66.2% for the chromosome III and 86% for the chromosome X). In contrast, centromeres of chromosome II did not associate strongly with any of the other centromeres (highest interaction at any stage was 20% with centromere III, Figure S2B). We thus concluded that the decrease in the number of CID foci (Figure 1) mainly resulted from homologous interactions, but that non-homologous interactions also contributed to this reduction.

### Synaptonemal components are expressed in the mitotic region and localize close to centromeres

**Figure 3:**
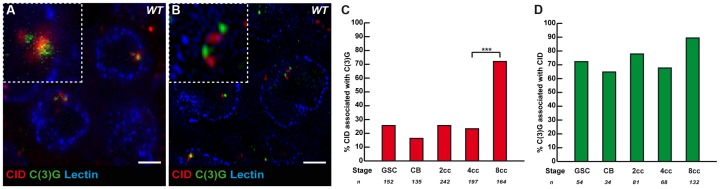
The Synaptonemal Complex component C(3)G is present near some centromeres in the mitotic region. (A) Projection of Z-sections obtained by confocal microscopy of a wild-type 2cc stained for centromere (CID, red), C(3)G (green) and nuclear membrane (Lectin, blue). Scale bar represents 2 µm. (B) Projection of Z-sections obtained by OMX of a wild-type 2cc stained for centromere (CID, red), C(3)G (green) and nuclear membrane (Lectin, blue). Scale bar represents 2 µm. (C) Developmental changes in the percentage of centromeres associated with C(3)G for each cell stage in region 1. CID and C(3)G are associated when the 2 foci touch or overlap in a nucleus. Cysts were staged according to the shape of the fusome marked by α-spectrin (not shown). (D) Developmental changes in the percentage of C(3)G associated with centromeres for each cell stage in region 1. CID and C(3)G are associated when the 2 foci touch or overlap in a nucleus. Cysts were staged according to the shape of the fusome marked by α-spectrin (not shown).

It is known that the formation of the SC initiates at the clustered centromeres as soon as cysts enter region 2, and that SC proteins are required for the clustering of centromeres in the same region [13]–[14]. Could SC components, such as C(3)G and Corona, also be required to initiate the pairing and clustering of centromeres in region1? C(3)G and Corona are transverse filaments and central element components of the SC, respectively, and are required for SC formation [18]–[19]. Surprisingly for meiosis-specific proteins, we found that C(3)G and Corona were expressed in germ cells of the mitotic zone and associated with centromeres [18]–[19] (Figure 3A, B and data not shown). No staining was detected in nuclei of either wild-type follicle cells or *c(3)G* mutant germ cells attesting to the specificity of the signal (data not shown). Using wide-field deconvolution microscopy and confocal microscopy, we found that C(3)G signals were partially overlapping with CID at centromeres (Figure 3A). However, using super-resolution microscopy, we could separate CID foci from C(3)G dots (Figure 3B). It showed that C(3)G localized very close to the centromeres, but not at centromeres, at least marked by CID. We further found an increase in the association of C(3)G with centromeres from GSCs to 8-cell cysts. In GSCs, 25.7% (n = 152) of centromeres overlapped or touched a C(3)G staining, while there was a dramatic increase in 8-cell cysts, where 72.0% of centromeres (n = 164) overlapped with dots of C(3)G (Figure 3C). Furthermore, we found that most dots of C(3)G in each nucleus were associated with centromeres at every stage (Figure 3D). The accumulation of C(3)G at centromeres correlates with an increase in centromere interactions, suggesting that SC components may play an active role in this process.

### Synaptonemal components promote centromere clustering in the mitotic zone

**Figure 4:**
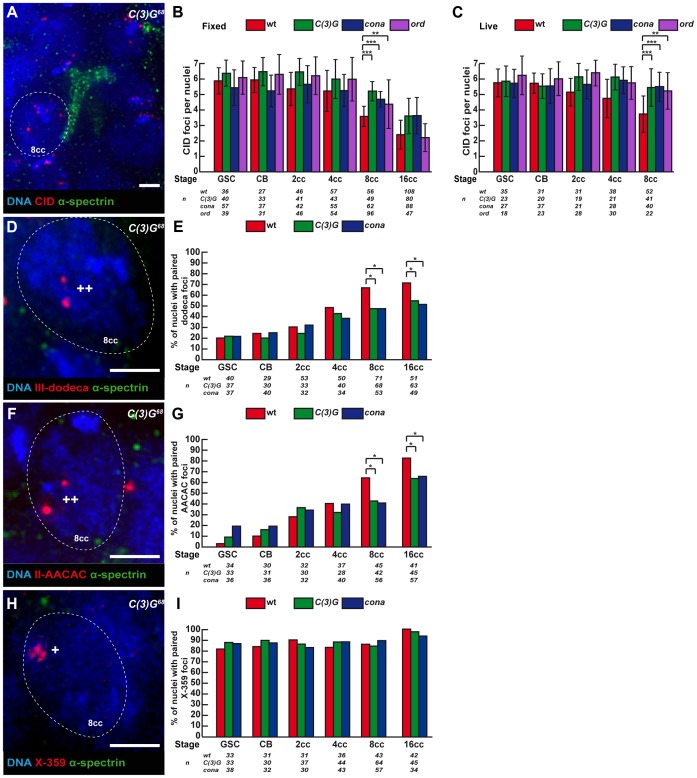
Synaptonemal Complex components promote centromere association in the mitotic zone. Fusome (α-spectrin, green) and DNA (DAPI, blue). (A) Projection of Z-sections obtained by DV microscopy of a *c(3)G^68^* germarium stained for centromere (CID, red), fusome, and DNA. One nucleus of an 8-cell cyst with a branched fusome is shown. Scale bar represent 2 um. (B,C) Developmental changes in the number of CID foci for each cell stage in region 1 in wild-type, *c(3)G^68^*, *cona^f04903^/cona^A12^*and *ord^5^/ord^10^* fixed germaria (B) or in living germaria (C). The number of analyzed cells is indicated under each stage. * p≤0.05, ** p≤0.005, *** p≤0.0005, (two-tailed Student's t-test comparing wild-type with *c(3)G^68^* and *cona^f04903^/cona^A12^*). (D) Projection of Z-sections obtained by DV microscopy of a *c(3)G^68^* germarium stained for dodeca (red), fusome , and DNA. One nucleus of an 8-cell cyst (8cc), surrounded by dotted lines, displays two foci separated by a distance ≥0,7 µm (++) indicating unpaired centromeres. Scale bar represents 2 µm. (E) Developmental changes in the percentage of paired chromosomes III for each cell stage in region 1 using the dodeca probe in wild-type, *c(3)G^68^* and *cona^f04903^/cona^A12^* fixed germaria. Chromosome III centromeres are considered paired when the distance between the 2 foci ≤0,7 µm in a nucleus. The number of analysed cells is indicated under each stage. * p≤0.05 (khi2 test comparing wild-type with *c(3)G^68^* and *cona^f04903^/cona^A12^*). (F) Projection of Z-sections obtained by DV microscopy of a *c(3)G^68^* germarium stained for AACAC (red), fusome, and DNA. One nucleus of an 8-cell cyst (8cc), surrounded by dotted lines, displays two foci separated by a distance ≥0,7 µm (++) indicating unpaired centromeres. Scale bar represents 2 µm. (G) Developmental changes in the percentage of paired chromosomes II for each cell stage in region 1 using the AACAC probe in wild-type, *c(3)G^68^* and *cona^f04903^/cona^A12^* fixed germaria. Chromosome II centromeres are considered paired when the distance between the 2 foci ≤0,7 µm in a nucleus. The number of analysed cells is indicated under each stage. * p≤0.05 (khi2 test comparing wild-type with *c(3)G^68^* and *cona^f04903^/cona^A12^*). (H) Projection of Z-sections obtained by DV microscopy of a *c(3)G^68^* germarium stained for X-359 (red), fusome, and DNA. One nucleus of an 8-cell cyst (8cc), surrounded by dotted lines, displays one focus (*) indicating pairing. Scale bar represents 2 µm. (I) Developmental changes in the percentage of paired X-chromosomes for each cell stage in region 1 using the 359 probe in fixed wild-type, *c(3)G^68^* and *cona^f04903^/cona^A12^*fixed germaria. Chromosome X centromeres are considered paired when the distance between the 2 foci ≤0,7 µm in a nucleus. The number of analyzed cells is indicated under each stage.

To test whether the presence of SC components at centromeres in region 1 had a function in centromere association, we counted the number of CID dots in *c(3)G^68^* and *cona^f04903^/cona^A12^* mutants in both live and fixed tissue. We found that the association of centromeres was dramatically affected in both mutant backgrounds at the 8-cell and 16-cell stages (Figure 4). *c(3)G* mutant 8-cell cysts show an average of 5.2±1.4 CID foci as compared to 3.6±1.3 in the wild-type situation (two-tailed Student's t-test, p<0,0005). Instead the number of CID dots in mutant 8-cell cysts is similar to wild-type GSCs (5.9±0.9) (Figure 4B, C). Furthermore, we never observed nuclei with only 1 or 2 dots of CID in mutant 8-cell cysts (0%, n = 49; Figure S1), indicating that C(3)G and Corona were also required for centromere clustering in region 1. We have analysed centromere pairing and clustering in *ord^5^/ord^10^* mutant females both on fixed and live germarium (Figure 4B and 4C). Ord is a cohesion protein, which localizes to the Lateral Elements (LEs) of the SC. We found that the number of CID foci was significantly higher in *ord* mutant females from GSC to 8-cell cysts, similar to *c(3)G* mutant (Figure 4B and 4C). However, the increase in CID foci at the 8-cell stage became weaker in *ord* mutant females than in *c(3)G* mutants (4.3±1.6 CID foci in *ord^5^/ord^10^* compared to 5.2±1.4 in *c(3)G^68^* and 3.6±1.3 in WT). By the 16-cell stage, centromeres were well clustered in *ord* mutant germaria (2.2±0.9 CID foci in *ord^5^/ord^10^* compared 2.4±0.9 in WT). Surprisingly, later on in region 2a, the number of CID foci rose dramatically again (data not shown), as published in (Takeo et al, 2011). In conclusion, *ord* mutant germ cells initially behaved like *c(3)G* and *corona* mutants, then were “rescued” in young 16-cell cysts, and finally centromeres separated again in region 2a.

We then investigated how the pairing of individual centromeres is affected in our mutants in different cysts by combining FISH with immunostaining against the fusome (Figure 4D, F, H). We found that in *c(3)G^68^* and *cona^f04903^/cona^A12^* mutant 8-cell cysts, the number of paired chromosomes *III* at the level of the centromeric regions dropped from 66% (wt, n = 71) to 47% (*c(3)G^68^*: n = 68, *cona^f04903^/cona^A12^*: n = 53; khi^2^, p<0,02) (Figure 4E); and for chromosome *II*, pairing went from 64% (wt, n = 45) to 42.9% (*c(3)G^68^*: n = 42; khi^2^, p<0,05) and to 41.1% (*cona^f04903^/cona^A12^*: n = 56; khi^2^, p<0,02) (Figure 4G). In contrast, *X*-chromosome centromere pairing is unaffected in both *c(3)G^68^* and *cona^f04903^/cona^A12^* mutant germaria (Figure 4H, I). Thus, the SC components C(3)G and Cona are important for pairing of chromosome *II* and *III*, but not for chromosome *X* pairing, at the level of the centromeric regions in the mitotic zone.

## Discussion

In most species, early meiotic prophase can be characterized by the pairing of homologous chromosomes, the appearance of synaptonemal complex components or the re-organisation of chromosomes in bouquet or similar conformations [5]–[6]. We found that all three processes initiate in the mitotic region of the *Drosophila* germarium and predominantly in 8-cell cysts, i.e. well before the onset of meiotic prophase. Our finding that centromeres of chromosome *II* and *III* are not paired in GSCs further indicates that “somatic” pairing *stricto sensu* does not occur in these cells. Thus, one can conclude that meiotic pairing is not an extension of somatic pairing present in GSCs. However, we note that most centromeres are paired and clustered in 8-cell cysts, indicating that the meiotic nuclear organization in region 2a, i.e. when centromeres are fully paired and clustered, can still be viewed as an extension of a pre-existing state. In an interesting twist, we show that pairing and clustering of centromeres in the mitotic region depend partially on meiotic proteins such as C(3)G and Corona. Therefore, although meiotic pairing is the continuation of mitotic pairing, we show that mitotic pairing in germ cells depends on several meiotic proteins.

We found an average of 6 foci of CID in GSCs, which can be explained by half of the centromeres being paired and the other half being unpaired. Centromeres of the X chromosome appear always paired, while centromeres of the chromosome *II* and *III* are mostly unpaired. It suggests that the remaining chromosome 4 is mostly paired. Further experiments are however required to demonstrate this hypothesis. In addition, we found some non-homologous interactions between centromeres of chromosome X and *III*. It is also known that chromosomes X and 4 tend to be associated in different contexts. We can thus speculate that centromeres of chromosome X, III and 4 are preferentially associated together when centromere clustering becomes complete in region 2a, while centromeres of chromosome II remain distinct. This model would be compatible with the average of 2 dots of CID observed in region 2a [13]–[14]. One large dot would correspond to centromeres of chromosome X, III and 4, while the smaller dot would often correspond to chromosome II centromere.

Our data show that chromosome *X* (and probably chromosome 4) behaves differently and appears always paired throughout oogenesis. This pairing is independent of C(3)G and Corona, and the underlying mechanisms remain to be identified. Since both *X*-chromosomes have centromeric clusters of rDNA repeats that localize to the same nucleolus, one could speculate that mechanisms similar to *Drosophila* males X-Y pairing may help pairing in this centromeric region [20]. It is likely that additional mechanisms also facilitate pairing of autosomal homologues in germline cysts. Indeed, although we found that centromeric pairing is dramatically affected in 4- and 8-cell cysts mutant for *c(3)G*, there remains a tendency to pair in region 1 and also later in region 2 (Figure S3 and [13]). In addition, the lab of Ting Wu (Harvard Medical School) found the same pairing behavior for the chromosome arms, as we did for centromeric regions (Eric Joyce, Nicholas Apostolopoulos, Brian J. Beliveau, and C.-ting (Ting) Wu, personal communication). Using oligopaints probes targeting euchromatic and heterochromatic sequences on each chromosome arm, they found that the chromosome X was always paired, and that chromosomes II and III only became paired later on during cyst divisions. We found that C(3)G and Corona localize only close to the centromeres and peri-centromeric regions. It remains unknown whether pairing along chromosome arms also depends on C(3)G and Corona. We can thus hypothesize that other mechanisms independent of C(3)G and Corona are also at play to promote pairing even for chromosomes *II* and *III*.

It also remains possible that C(3)G and Corona do not play a direct role in promoting homologous pairing for chromosome *II* and *III*. These SC components could be mainly required to facilitate centromere association both homologous and non-homologous, which would indirectly increase the efficiency of homologous pairing. In support of this view, it is interesting to note that the 20% of 8-cell cysts with fully clustered centromeres (1 or 2 dots of CID, Figure S1A, S1E) disappeared in the absence of C(3)G (Figure S1B, S1F), indicating that C(3)G is absolutely required for clustering of centromeres. This absence of clustering could account for the 30% decrease in homologous pairing in *C(3)G* mutant (Figure 4). In this model C(3)G and Corona would not have a role in centromere homology assessment, and an independent homolog pairing process would act in parallel.

Our results in *Drosophila* reveal surprising similarities with the initiation of meiosis in budding yeast. Indeed, centromeres in *S. cerevisiae* also become associated or “coupled” before meiotic prophase [21]. This early association depends on Zip1, a central component of the SC functionally similar to C(3)G and SYCP1 in mouse. This similarity with our results further extend to the localization of Zip1, which partially overlaps with yeast centromeres at this early stage, like C(3)G and CID in flies [21]. The resemblance between C(3)G and Zip1 localization and function is surprising, as it is well known that SC formation requires double strand breaks (DSBs) in yeast but not in *Drosophila* [22]–[24]. Pre-meiotic pairing of homologues is thus present in a wide range of organisms, although the underlying mechanisms remain unknown in most cases [25]. Our results open the possibility to test whether the molecular mechanisms identified here in flies facilitate pre-meiotic pairing in other organisms.

## Materials and Methods

### Fly stocks and genetics

For all experiments on fixed germaria the following strains were used: *w^1118^* was used as the wild-type strain. For testing meiotic mutants, the following null alleles were used: *c(3)G^68^ e ca/c(3)G^68^ e* [18], [26], *cona^A12^/cona^f04903^* [19], *ord^5^/ord^10^* [27]. For experiments on live germaria CID::RFP [28], *nos*-Gal4, UASp::Par1:GFP/+ (gift from Daniel St Johnston) was used as a wild-type strain. Similarly the following mutant strains were used: CID::RFP, *c(3)G^68^ e/*Par1::GFP, *c(3)G^68^ e ca* and CID::RFP, *cona^A12^/*Par1-GFP, *cona^f04903^*.

### Immunohistochemistry and FISH

For immunostaining, ovaries were dissected in PBS, fixed in 4%PFA-PEPS, permeabilized in PBT (0,2%Triton) for 30 min, left overnight with primary antibodies in PBS at 4°C , washed 4×30 min in PBS, left with secondary antibody for 2 hr at room temperature, washed 4×30 min in PBS where DAPI (1∶500) was added during the last wash and mounted in Cityfluor. For FISH experiments followed by immunostaining, ovaries were dissected in PBS fixed in 4% PFA in 1× fix buffer (100 mm potassium cacodylate, 100 mm sucrose, 40 mm sodium acetate, and 10 mm EGTA). Ovaries were then rinsed three times in 2× SSCT and hybridization with the dodeca and 359 probes targeting the pericentromeric regions of the 3^rd^ and X chromosome respectively was performed as previously described [11]. Following hybridization ovaries were rinsed in 2× SSCT, rinsed two times in PBST and the immunostaining protocol was resumed as above. We used the following primary antibodies: mouse anti-c(3)G (1/500) (gift from Scott Hawley, Stowers Institute, USA), rat anti-cid (1∶1000) (gift from Claudio E. Sunkel, Universidade do Porto, Portugal). We used rabbit anti-spectrin (1∶1000 for regular immunostaining, 1∶500 following FISH) (gift from R. Dubreuil) to label the fusome and identify the cyst stages. Secondary antibodies conjugated with Cy3, Cy5 and FITC (Jackson laboratories) were used at 1/200.

### Image acquisition and data analysis

DV Images of fixed germaria were collected under a DeltaVision deconvolution microscope system (Applied Precision) equipped with an Olympus 1670 inverted microscope and high-resolution CCD camera. All images were acquired with the PlanApo 60×/1,42 oil objective lens with 1.5× auxiliary magnification at 0.2 µm intervals along z-axis and deconvolved using the softWoRx v.3.5.1 software (Applied Precision).

Confocal Images of fixed germaria were collected under a Zeiss LSM 780 NLO confocal. All images were acquired with a PlanApo 63×/1,46 oil objective at 0.6 um along z-axis and operated by ZEN 2012 software.

When structured illumination was performed we used an OMX v3 system (Applied Precision- gehealthcare), equipped with 3 EMCCD, evolve cameras (photometrics). Signal from all channels were realigned using fluorescent beads prior to each session of image acquisition. Registration was done using UnwarpJ in ImageJ. All images were acquired with a PlanApo 100×/1,4 oil objective at 125 nm along z-axis. Pixel size is 40 nm along xy-axis after reconstruction.

For live imaging ovaries were dissected in oil (10S, Halocarbon, Sigma). The muscular sheath around each ovariole was removed and germaria were made to stick to coverslips in oil.

Movies were collected with an Inverted Spinning disk Confocal Microscope (Roper/Nikon) operated by Metamorph on an inverted Nikon Eclipse T*i* microscope coupled to a CoolSNAP HQ2 camera (Photometrics) and temperature control chamber. All images were acquired with the Planapo 60×/1,4 oil objective lens with 1.5× auxiliary magnification. Single position movies in the germarium were acquired for 30–40 min at 25±1°C, with a 30 sec temporal resolution (7 slices *Z*-stack, 0.7 µm/slice).

The use of α-spectrin and Par1:GFP on fixed and live germaria respectively allowed to precisely identify the different cyst stages. For quantification of CID foci on fixed germaria, we counted the number of distinguishable CID foci within each single nucleus, and for live germaria we took into account the number of distinguishable CID foci at a given (t) projection where the number of CID foci was maximal. In all figures the images of fixed germaria shown are the projection of all z-series which cover a region ranging from the first CID foci until the last CID foci seen. For live germaria images shown are the projection of all z-series of a single (t) projection.

To measure the distances between foci of the dodeca and 359 probes a macro designed by Olivier Leroy from the unit imaging facility was used that computes 3D distances between selected foci. Peri-centromeric regions of chromosomes were considered as paired when only one foci was visible or when two foci were visible but separated by a distance ≤0,70 µm [10].

## Supporting Information

Figure S1 Synaptonemal Complex components promote centromere pairing in region 1. Distribution of the number of CID dots per stage in wild-type (A, E), *c(3)G^68^* (B, F), *cona^f04903^/cona^A12^* (C, G) and *ord^5^/ord^10^* (D, H) in fixed (A–D) and in living (E–H) germaria. Each color corresponds to a number of CID dots per nuclei as defined on the panel.
(PDF)

Figure S2 Non-homologous centromere interactions in the mitotic zone. (A) Projection of Z-sections obtained by DV microscopy of a wild-type germarium stained for the chromosome III centromere (dodeca probe, red), the chromosome II centromere (AACAC probe, green), the fusome (α-spectrin, white), and DNA. 2 nuclei of an 8-cell cyst with weak fusome staining are illustrated as examples. In the Nucleus with * a heterologous association between a chromosome II centromere (green) and a chromosome III centromere (red) is seen. In the Nucleus with ++ no heterologous associations are seen. Note that fusome immunostaining after FISH sometimes results in non-specific aggregates as seen in the Nucleus with ++. Scale bar represents 2 µm. (B) Developmental changes in the percentage of chromosome III centromeres interacting with either chromosome X centromeres (light blue columns) or chromosome II centromeres (red columns) for each cell stage in region 1 in fixed wild-type germaria. (C) Developmental changes in the percentage of chromosome II centromeres interacting with either chromosome X centromeres (green columns) or chromosome III centromeres (dark blue columns) for each cell stage in region 1 in fixed wild-type germaria. (D) Developmental changes in the percentage of chromosome X centromeres interacting with either chromosome II centromeres (violet columns) or chromosome III centromeres (yellow columns) for each cell stage in region 1 in fixed wild-type germaria. The number of analyzed centromeres is indicated under each stage.
(PDF)

Figure S3 Developmental changes in the number of CID foci for each cyst stage in region 1 in wild-type (A), *c(3)G^68^* (B), *cona^f04903^/cona^A12^* (C) and *ord^5^*/*ord^10^* (D) in fixed germaria. The number of analyzed cells is indicated under each stage. *** p≤0.0005 (two-tailed Student's t-test comparing 4cc with 8cc and 8cc with 16cc).
(PDF)

Movie S1 Dynamics of centromeres clusters in region 1. Time lapse microscopy (spinning disc) of a living CID::RFP/+; nos>Par1::GFP/+ germarium expressing the centromere marker CID::RFP (red) and the fusome marker Par1::GFP (green). Two germinal stem cells (GSC) are identified by their position close to the niche and their spectrosome. Two cystoblasts (CB) are identified by their round fusome, and a 4-cell cyst (4cc), whose cells are linked by a typical U-shaped fusome, demonstrating that they are from the same cyst. The localization of two 8-cell cysts (8cc), with a faint fusome GFP signal, is indicated. Arrow points towards moving clustered centromeres in a 4cc. An enlargement at t = 6∶30 min shows the maximum number of CID foci counted for this 4cc.
(AVI)
