# Supplementary figures and images for: Synaptonemal Complex Components Promote Centromere Pairing in Pre-meiotic Germ Cells
Source: PLoS Genet. 2013 Dec 19;9(12):e1004012. doi: 10.1371/journal.pgen.1004012 (PMC3868581; doi:10.1371/journal.pgen.1004012)

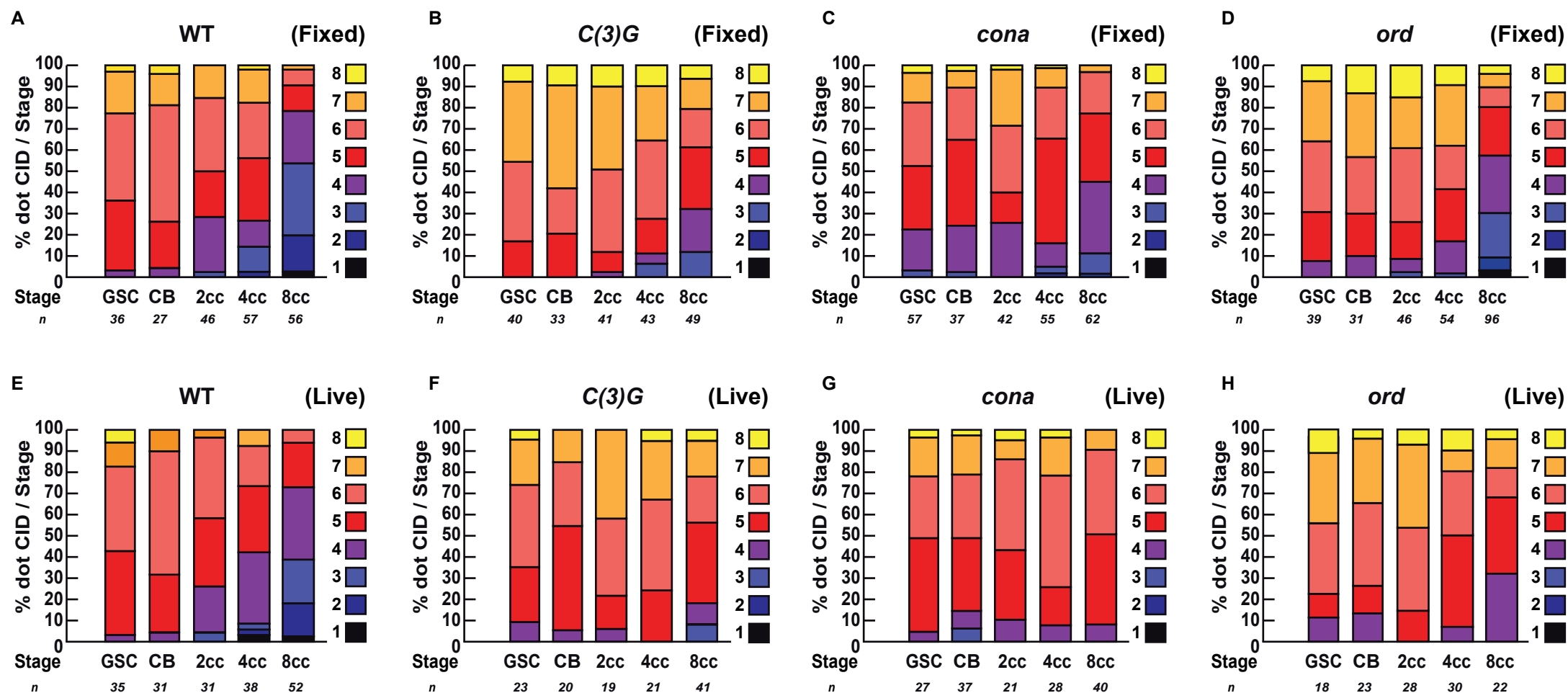

Figure S1

Supplement: Figure S1 — Synaptonemal Complex components promote centromere pairing in region 1. Distribution of the number of CID dots per stage in wild-type (A, E), c(3)G68 (B, F), conaf04903/conaA12 (C, G) and ord5/ord10 (D, H) in fixed (A–D) and in living (E–H) germaria. Each color corresponds to a number of CID dots per nuclei as defined on the panel. (PDF) [file pgen.1004012.s001.pdf]

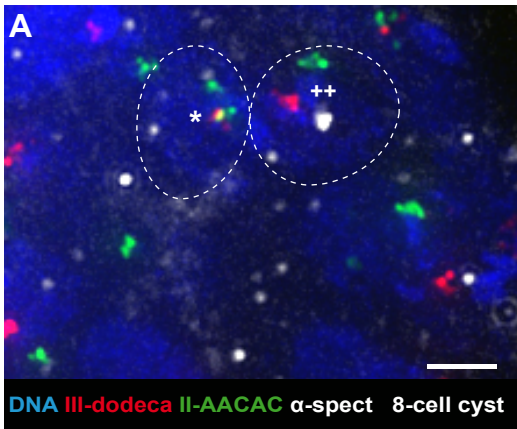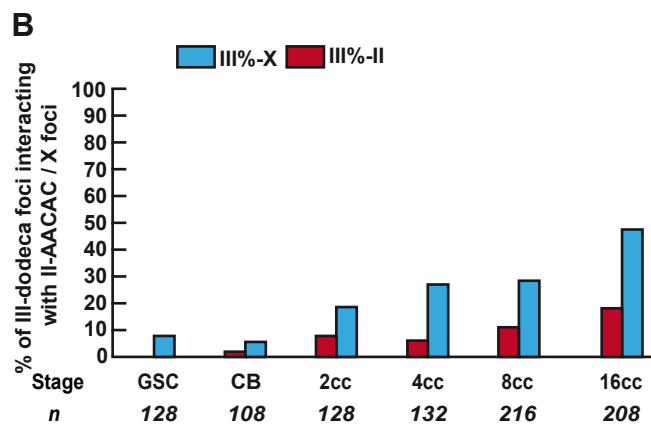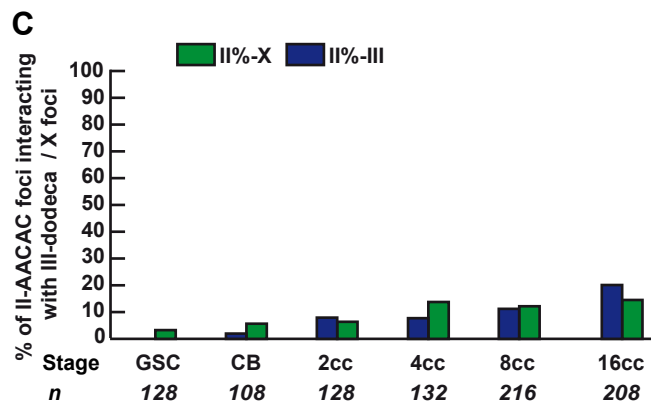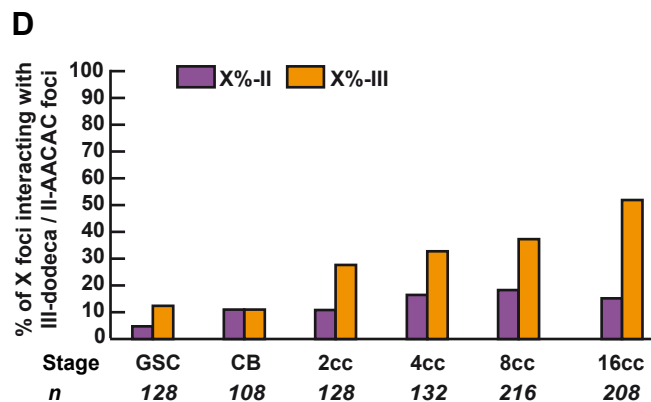

**Figure S2**

Supplement: Figure S2 — Non-homologous centromere interactions in the mitotic zone. (A) Projection of Z-sections obtained by DV microscopy of a wild-type germarium stained for the chromosome III centromere (dodeca probe, red), the chromosome II centromere (AACAC probe, green), the fusome (α-spectrin, white), and DNA. 2 nuclei of an 8-cell cyst with weak fusome staining are illustrated as examples. In the Nucleus with * a heterologous association between a chromosome II centromere (green) and a chromosome III centromere (red) is seen. In the Nucleus with ++ no heterologous associations are seen. Note that fusome immunostaining after FISH sometimes results in non-specific aggregates as seen in the Nucleus with ++. Scale bar represents 2 µm. (B) Developmental changes in the percentage of chromosome III centromeres interacting with either chromosome X centromeres (light blue columns) or chromosome II centromeres (red columns) for each cell stage in region 1 in fixed wild-type germaria. (C) Developmental changes in the percentage of chromosome II centromeres interacting with either chromosome X centromeres (green columns) or chromosome III centromeres (dark blue columns) for each cell stage in region 1 in fixed wild-type germaria. (D) Developmental changes in the percentage of chromosome X centromeres interacting with either chromosome II centromeres (violet columns) or chromosome III centromeres (yellow columns) for each cell stage in region 1 in fixed wild-type germaria. The number of analyzed centromeres is indicated under each stage. (PDF) [file pgen.1004012.s002.pdf]

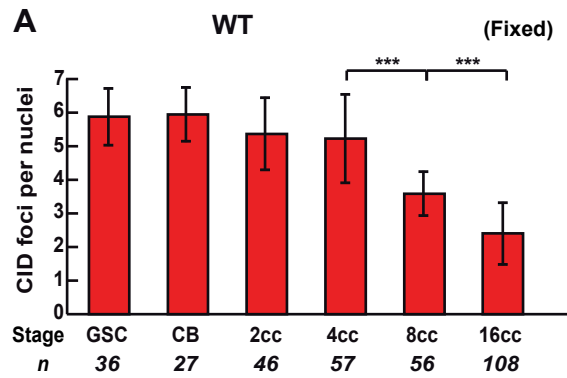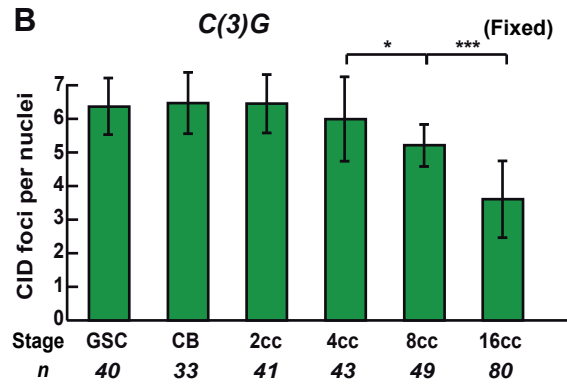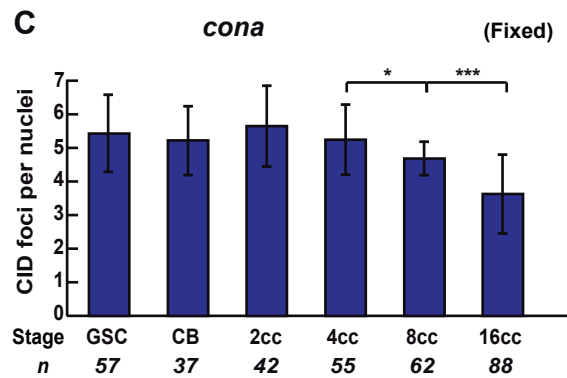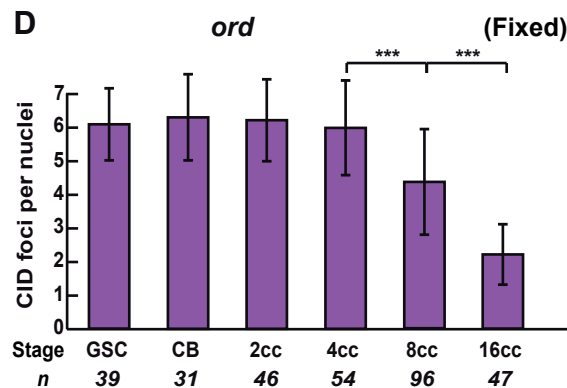

Figure S3

Supplement: Figure S3 — Developmental changes in the number of CID foci for each cyst stage in region 1 in wild-type (A), c(3)G68 (B), conaf04903/conaA12 (C) and ord5/ord10 (D) in fixed germaria. The number of analyzed cells is indicated under each stage. *** p≤0.0005 (two-tailed Student's t-test comparing 4cc with 8cc and 8cc with 16cc). (PDF) [file pgen.1004012.s003.pdf]
